# Linking Social Cohesion to Biological Markers of Aging: Evidence from NHATS

**DOI:** 10.1093/geroni/igaf122.787

**Published:** 2025-12-31

**Authors:** Tara Klinedinst, Nicholas Hollman, Michael Hankes, Raymond Jones

**Affiliations:** The University of Oklahoma Health Sciences Center, Oklahoma City, Oklahoma, United States; The University of Oklahoma School of Community Medicine, Tulsa, Oklahoma, United States; The University of Alabama at Birmingham, Birmingham, Alabama, United States; The University of Alabama at Birmingham, Birmingham, Alabama, United States

## Abstract

Interest in the complex intersection between social determinants of health and biological aging markers is growing. Social cohesion, a measure of community belonging, and inflammatory biomarkers, like interleukin-6 (IL-6) and c-reactive protein (CRP), have emerged as significant factors influencing age-related chronic conditions and functional decline among older adults. With data from the National Health and Aging Trends Study (NHATS), we used logistic regression to assess the relationship between sociodemographic variables and low social cohesion, as well as multivariable linear regression to test the association between low social cohesion and IL-6 and CRP. In our sample of 4,648 older adults, the average age was 76 years old, majority female (55%), and non-Hispanic White (81%). We found that low social cohesion was associated with minority status, lower income, higher BMI, >3 chronic conditions, probable dementia, frailty, depression, and lack of walking for exercise. Additionally, low social cohesion was associated with higher levels of CRP and IL-6 after adjusting for the influence of sociodemographic variables. Understanding the link between social cohesion and inflammation is particularly relevant in aging populations, as with age, social networks often diminish, and the prevalence of inflammatory conditions increases. This research provides critical insights into the potential benefits of improved social engagement and feelings of community, and the relationship to inflammation and other health outcomes among older adults. These findings establish a target for health policy and future intervention research, namely improving health outcomes through a specific focus on improving social cohesion and community.

